# A CAP for Healthy Living Mainstreaming Health into the EU Common Agricultural Policy: European Public Health Alliance (EPHA), 2015

**DOI:** 10.3934/publichealth.2015.4.844

**Published:** 2015-12-18

**Authors:** Nikolai Pushkarev

## Strategic Objectives

Agriculture and health are intimately connected. Good food is essential for a healthy life. The Common Agricultural Policy (CAP) was successful in tackling food insufficiency in post-war Europe. However, despite numerous reforms, it fails to meet the public health challenges of today. Unhealthy diets, smoking and harmful alcohol consumption are key causes of chronic diseases, responsible for the overwhelming majority of Europe's burden of mortality (86%) and disease (77%). Environmental degradation, antibiotic resistance and climate change endanger the last century's significant gains in health. Agriculture is implicated in all of these.

This report presents observations and recommendations on ways to enhance coherence between the CAP and public health, expressed through a number of strategic objectives:

**Ensure full application of the Health in All Policies obligation**

See sections: “Include public health as a CAP objective”; “Market organisation must not restrict health”

**Align the CAP with promoting sustainable diets**

See sections: “No cheap sugar”; “Rebalance the supply of livestock products”; “Boost vegetables and fruit production & consumption”; “Promote products for diverse and healthy diets”; “There is no such thing as cheap food”

**Make the CAP consistent with reducing harmful alcohol consumption**

See sections: “No public money for wine overproduction”; “No need to support hops”

**Make the CAP consistent with reducing tobacco use**

See section: “End all subsidies for tobacco cultivation”

**Build a CAP that enhances delivery of public goods**

See sections: “Transition towards forward-looking direct payments”; “Support transformative rural development”

At the same time, this paper realises the limits of the CAP as an instrument to improve public health outcomes. The food and beverage processing and retail industries, in the context of international supply chains, are now the key actors in shaping people's diets, farmers' incomes and production decisions. Therefore the food system ought to be addressed as a whole – from farm to plate – in a coherent, health-sensitive and sustainable European food and agricultural policy.

## A CAP for Healthy Living

“The Common Agricultural Policy of the European Union is not about farmers.

It is about food and it is about nutrition – it's about healthy living.”

Phil Hogan, EU Commissioner for Agriculture [1]

The Common Agricultural Policy (CAP) was largely introduced as a public health measure. Conceived during post-war reconstruction while Europeans still feared food shortages and disruptions in supply, the CAP's original focus was to increase food sufficiency, which, it was assumed, would automatically lead to health[2]. The policy's preoccupation with increased production and productivity was a logical outcome of this vision, and judged by this measure the CAP succeeded remarkably well[3].

Contemporary public health concerns are very different from the ones the CAP encountered during its inception and its original approach unsuitable for the challenges of unhealthy diets, smoking and harmful alcohol consumption faced today[4]. Admittedly, the CAP went through a wave of reforms from the 1990s onwards, gradually shifting the policy away from production subsidies to producer support, a transition that helped reduce some of the glaring health inconsistencies. Public health considerations however never drove these policy changes and this can explain why various measures in the CAP are still incompatible with public health and the ‘Health in All Policies’ obligation enshrined in the European Treaty[5].

Just as European Commissioner for Agriculture Phil Hogan suggested in the opening quote above, public health should regain its place at the heart of European Union (EU) agricultural policy. A public health approach to the CAP will not only help reduce preventable chronic diseases and their vast societal consequences, but will also alleviate the economic and ecological challenges that the agricultural sector faces. It will furthermore justify the survival of the CAP, which is under constant pressure.

EPHA also argues that the CAP is only the first step to address the health impacts of the food system at large. The processing and retail industries, unlike when the CAP was instituted, are now the key actors in shaping people's diets and farmers' incomes and production decisions. Therefore today, public policy based on good governance and budgetary responsibility can be achieved only when the food system is addressed as a whole – from farm to plate – in a coherent, health-sensitive and sustainable European food and agricultural policy.

### Agriculture and health

Agriculture is linked to all the major causes of mortality and disease in the European Union (EU). Together with smoking, harmful alcohol consumption and physical inactivity, diets are responsible for the overwhelming majority of ill-health in Europe[6]. Unhealthy diets are the single biggest risk factors for disability adjusted life years lost (DALYs) in the EU[7],[8].

**Figure:**
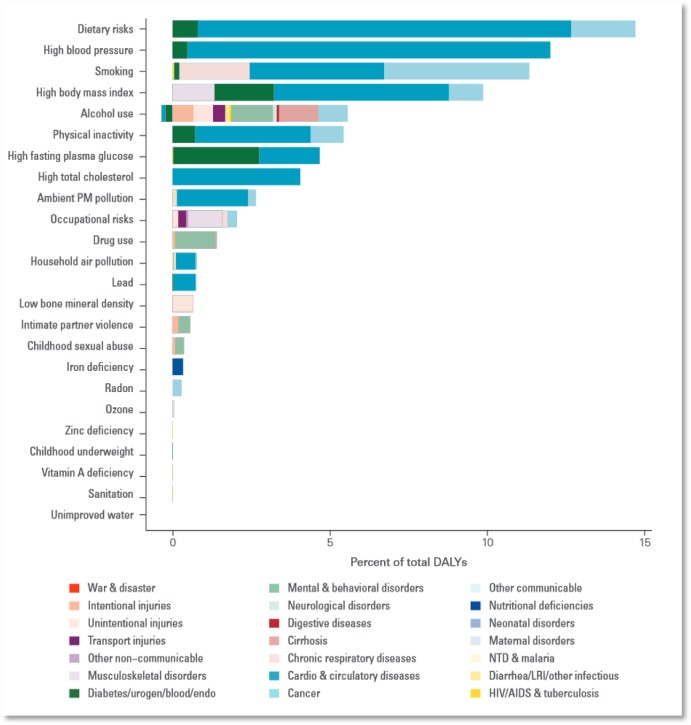
Disease adjusted life years lost by risk factors, EU and EFTA, 2010 (The Global Burden of Disease)

The graph above shows the main risk factors for DALYs in the EU. The top risk factors are easily linked to agriculture. Alcohol and tobacco are based on agricultural raw materials. Dietary risk, high blood pressure, high body mass index, high blood glucose and high cholesterol are all directly linked to food and dietary practices, which are influenced by factors like food availability and relative prices. In addition to the impacts on the food chain, agriculture also influences health through the farming methods employed. Livestock raising is for instance an important contributor to ambient particulate matter (PM) pollution. Even physical activity levels can be partly related to urbanisation and changes in agricultural production technology.

#### Health sensitive products

Agriculture is not ‘just’ another sector of the economy producing ‘mere’ commodities. Agriculture produces food, a basic human need and key determinant of life quality. But agriculture also produces more than that. The socio-economic and technological structures of the agricultural sector shape rural communities, urbanisation rates, employment opportunities, farming cultures, the quality of landscapes, nature and biodiversity, ecosystem services, animal welfare and the climate.

From a health perspective not all kinds of agricultural products are the same and neither are all production methods. Some agricultural products are more ‘health sensitive’ than others. The clearest example is the tobacco crop whose sole widespread use is to be transformed into tobacco products, which kill 700,000 people in Europe every year [9]. Several other products can similarly be categorized as health-sensitive, like wine grapes, sugar, some livestock products, hops and vegetables and fruits.

This report focuses on health-sensitive products and production practices and will discuss the CAP's provisions from the perspective of health-based strategic aims. This report is the first within a series of papers exploring the links between different parts of the food system and health.

**Sustainable diets**

One of the strategic aims of this paper is to align the CAP with efforts to promote sustainable diets. What are sustainable diets and why should sustainability matter to public health?

The Food and Agricultural Organisation of the United Nations (FAO) defines sustainable diets as[10]:

“Sustainable Diets are those diets with low environmental impacts which contribute to food and nutrition security and to healthy life for present and future generations. Sustainable diets are protective and respectful of biodiversity and ecosystems, culturally acceptable, accessible, economically fair and affordable; nutritionally adequate, safe and healthy; while optimizing natural and human resources.”

The links between public health and the environment are as old as the discipline of public health itself[11]. The validity of this association is confirmed by a growing body of evidence showing that human health is intrinsically linked to the health of the environment. On this basis The Lancet Commission on Planetary Health concludes that “*the continuing degradation of natural systems threatens to reverse the health gains seen over the last century*”[12]. Similarly, The Lancet Commission on Health and Climate Change says:

"The implications of climate change for a global population of 9 billion people threatens to undermine the last half century of gains in development and global health." [13]

This link is starting to be addressed as well in practice. For instance, the Finnish Nutrition Recommendations from 2014 pioneer with their inclusion of sustainability considerations in dietary recommendations[14]. In the US this inclusion was recently considered as well but, presumably due to pressure from influence groups, subsequently dropped[15].

### The CAP matters

The CAP is one of the most important EU policies, responsible for around 40% of the EU budget or approximately €57 billion per year [16]. Expenditure of such considerable resources has repeatedly come under scrutiny and calls are voiced for the policy to enhance delivery of public goods. The lion's share of ill-health and death are attributable to preventable diseases with diet, alcohol consumption and tobacco as core risk factors. Healthcare costs amount to 9% of European GDP[17],[18]. In a recent statement by the European Commissioner for Health and Food Safety Dr. Vytenis Andriukaitis, it is however the risk factors, most importantly diet, alcohol and tobacco use, rather than investments in healthcare services that result in the real economic costs to society[19].

In this light, public policy matters. Incentives provided by policies should signal a coherent vision of societal priorities. Expenditure in one sector of the economy, such as agriculture, should not result in external costs borne by other parts of the economy, such as the environment or public health. This basic principle is essential for maintaining the credibility of EU public governance in general and a support system like the CAP, whose very existence is called into question.

#### CAP and diets

But to what extent is the CAP responsible for dietary outcomes? This question is difficult to quantify given sparse research in the field[20]. Several reports, starting with the ground-breaking study by the Swedish National Institute of Public Health, have argued that there is a close link between the CAP and dietary choice[21]. A report by the Faculty of Public Health in the UK also eloquently argues for a direct connection between CAP budgetary priorities and diets[22].

**Figure:**
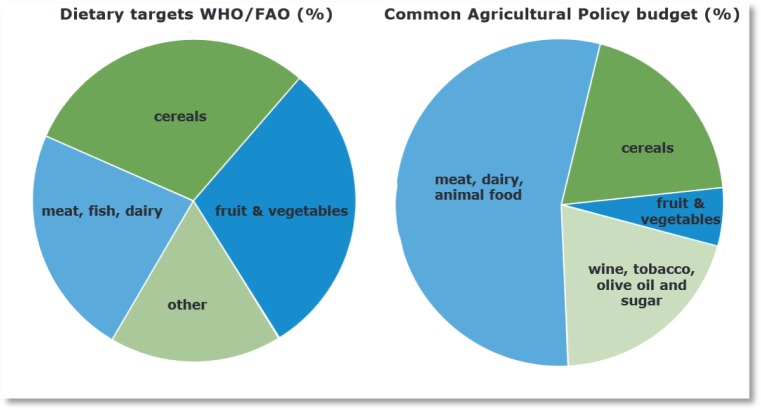
Contrast between dietary targets and CAP budget (Faculty of Public Health)

These reports are supported by research concluding that increases in food energy supply, in other words food availability, are the dominant drivers of weight gain in populations[23]. Investigations have shown that the rise in food energy supply can help explain increases in body weight in high income countries[24], the surge in overweight and obesity in the USA from the 1970s[25] and in the UK since the 1980s[26]. A related hypothesis implies that increased food supply pushes up population energy intake once an ‘energy balance tipping point’ is reached. Food consumption is then no longer driven by energy expenditure, but by the availability of food. This would be another strong argument for reconsidering the imperative of production and productivity growth in agriculture. It rather invites to shift focus to the question of *which* production should increase and *where[27]*.

**Figure:**
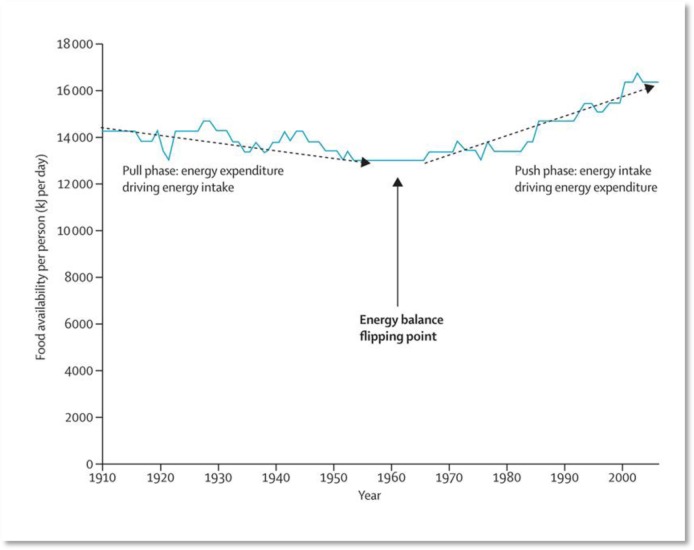
Food availability for the USA, 1910–2006 (Boyd Swinburn et al., The Lancet)

At the same time, other authors maintain that the CAP and dietary intakes are only faintly interconnected and that the CAP has effectively ‘taxed’ consumption of products like sugar, meat and milk by keeping European prices above the world market[28]. This is an important observation from agricultural economics. It misses the point however that from a consumers' perspective food affordability is more usefully measured against income than world prices. Besides, studies have shown that EU export subsidies helped to keep world prices artificially low for a number of key products[29]. Over recent decades the share of disposable income spent on food by consumers has fallen significantly, now at an average of less than 15% of total expenditure[30]. According to the OECD, increased affordability of food is one of the factors responsible for the rise of obesity[31]. Falling relative prices of food may have contributed to up to 40% of the increase in body mass index in the United States in the period 1976 to 1994[32].

This report is predicated on the evidence that a link between the CAP and dietary outcomes does exist. The policy's payment schemes and market organisation rules provide incentives to producers who react by adjusting production decisions, thereby influencing food availability and the general ‘food environment’ in Europe. Although trade in agricultural products is important from an economic perspective, only 11% of the total supply of primary agricultural products is imported from outside the EU and only 7% of the total output of the EU food, drink and tobacco sectors is exported[33]. This means that roughly 85–90% of the food Europe produces remains within the EU and is consumed or withdrawn in some way internally.

Yet it is also clear that the CAP, given the powerful position of the food processing and retail industries, is an imprecise and insufficient instrument alone to steer consumption patterns[34]. This underlines the need to take a Health in All Policies approach to the entire food system by integrating production, processing, wholesaling, retailing, trade, marketing and consumption into a consistent policy framework: a sustainable food and agricultural policy[35].

## Mainstreaming Health into the CAP: Observations & Recommendations

“Growing, buying and eating the right kinds of food can reduce the risk of disease and simultaneously promote a sustainable environment.”

WHO Europe[36]

This report presents observations and preliminary recommendations on ways to achieve coherence between the EU Common Agricultural Policy (CAP) and strategic public health objectives. The report addresses the new CAP, which entered into force in 2015, and aims to create a dialogue ahead of the mid-term review and the next CAP reform in 2020 to ensure the policy instruments and funding priorities are better able to deliver health for EU citizens.

Realising the multifunctional nature of agriculture, EPHA is not calling for an abolition of farm support or the slashing of all market regulations[37]. ‘The market’ without a proper regulatory framework would inevitably undervalue the key public goods that farmers can produce and reduce food to a commodity. In contrast, Europe needs a sustainable agricultural sector, vibrant rural communities supported by a policy that explicitly promotes health and overcomes the ‘lock-ins’ and path dependencies created by decades of ‘productivist’ incentives[38].

In this report EPHA addresses CAP spending and market rules in order to orient the policy towards producing more public value for public investment. Policy recommendations to the core three CAP instruments are considered:

Regulation 1307/2013- Direct Payments Regulation^[39]^Pillar IRegulation 1308/2013- CMO Regulation^[40]^Pillar IRegulation 1305/2013- Rural Development Regulation^[41]^Pillar II

### Promote public health as a CAP objective

In any assessment of European Union (EU) policies it is worthwhile recalling what the EU stands for. The EU's basics are codified in its two founding treaties: the Treaty on European Union (TEU) and the Treaty on the Functioning of the European Union (TFEU)[42]. Both are explicit in the primacy accorded to human health and well-being.

In the first paragraph of Article 3 TEU, which sets the very core of EU's objectives, we find:

“*The Union's aim is to promote peace, its values and the well-being of its peoples*”

Article 9 TFEU states:

“In defining and implementing its policies and activities, the Union shall take into account requirements linked to the promotion of a high level ... of human health.”

Article 168 TFEU, containing the explicit ‘health in all policies’ obligation, reads:

“A high level of human health protection shall be ensured in the definition and implementation of all Union policies and activities.”

The Treaty is clear on the obligation to ensure that all EU policy actions (and inactions) are based on public health as a core determinant. Unfortunately, this is not the case in practice. The CAP, as one of EU's most ‘dominant’ and oldest policies was established prior to the inclusion of the Health in All Policies provision into the Treaties. To ensure its application in practice, more legal incentives are required to guarantee proper attention to public health. Public health should be included among the CAP's explicit objectives, which are set out in Article 39 TFEU.

**Recommendation: Include the promotion of public health as one of the CAP's core objectives, with a special focus on the attainment of sustainable diets.**

The inclusion of public health could be phrased along the lines of: “The objectives of the common agricultural policy shall be: ... (f) To facilitate and not to counteract the attainment of high levels of human health within the Union as well as beyond its borders and in particular to promote the attainment of healthy sustainable diets.”

#### Health impact assessment

Including a public health impact assessment at each stage of policy development would operationalise public health within the CAP. Experience with one of the first national health impact assessments (HIA) of agricultural policy conducted in Slovenia showed that the HIA is a useful mechanism for raising broader public health issues onto the agricultural agenda[43]. The current public health priority in agriculture remains food safety, while in terms of the burden of disease food security, nutrition and other production-related health concerns are more important. While HIA of agricultural policy is a complex task, methodologies for such assessments exist and implementation could be carried out by qualified public health experts.

**Recommendation: Conduct a thorough public health impact assessment at each relevant stage of policy development within the CAP. The scope and methodology of assessment should be established in coordination with public health experts.**

### Market organisation must not restrict health

The Common Market Organisation (CMO) Regulation lays down market organisation and intervention rules for most agricultural subsectors. The Regulation does not explicitly address public health, although health is mentioned in several non-core provisions. Market rules have however been abused to restrict Member State's action to protect and improve public health. This undermines efforts to address the negative societal effects of health problems, including the vast economic and social burden placed on Member States' economies and health systems due to chronic diseases, for which the European Commission is developing a political action framework[44].

An ongoing court case illustrates this incoherence. The Scotch Whisky Association, together with other alcohol industry groups [45], have sought to abolish a Scottish law on minimum unit pricing (MUP) of alcohol on the grounds that they claim it to undermines the market organisation of wine and particularly the principle of free price formation. The alcohol industries launched legal proceedings against the law after its adoption, arguing there is no place for national legislation once market organisation rules are fully harmonised at EU level.

Case law dealing with a previous version of the CMO is quoted:

“*Where there is a regulation of the common organisation of the market in a given sector, the Member States are, according to settled case-law, under an obligation to refrain from taking any measures which might undermine or create exceptions to it*”[46].

In its submission to the ECJ the European Commission argued that if MUP were to be adopted in various Member States it would undermine the legislative assumptions on which the CMO Regulation is based. This shows a major inconsistency within the Commission and is in stark contradiction to the Commission's declarations regarding the primacy of national decision-making powers on health and to Commissioner Andriukaitis' recent remarks in support of minimum unit pricing of alcohol[47].

The case is now pending at the European Court of Justice (ECJ) for a preliminary ruling[48]. ECJ Advocate General Yves Bot concluded in his Opinion that the “*objectives of the CAP ‘cannot disregard’ requirements relating to the public interest … and that the protection of health ‘contributes to the attainment of objectives of the [CAP]’*”, but Member States must ensure the introduced measure can be justified on the ground of Article 36 TFEU, involving a test of proportionality[49].

The final ruling will likely have significant implications for the ‘room for manoeuvre’ available for national and regional authorities to protect and improve public health. It will likewise set a precedent with repercussions beyond alcohol. Regardless of the outcome of the case, it is unacceptable that market organisation measures such as those contained in the CMO could be abused by vested interests to strike down legitimate, democratically approved public health measures.

**Recommendation: Include a binding provision into the CMO Regulation to reiterate the status of public health in the EU Treaties and stipulate that nothing in the CMO Regulation may prevent national public authorities from taking legislative or other policy measures to protect and improve public health.**

The Regulation should clarify that: “nothing in the CMO Regulation should prevent Member States from taking evidence-based legislative or non-legislative measures for the protection and improvement of public health, including, but not limited to the introduction of fiscal and economic instruments, marketing policies, labelling requirements, point of sale measures and nutritional standards”.

### No public money for wine overproduction

Europe is the heaviest drinking region in the world. Harmful alcohol use is the fifth leading cause of death and disability worldwide[50], representing a monetary cost of €155.8 billion in 2010 in the EU[51]. Any policy that directly or indirectly contributes to alcohol overconsumption is counterproductive for public health. The CAP has for decades intervened in the wine market, both stimulating and trying to limit structural oversupply[52],[53]. Today, while we no longer speak of wine lakes, overproduction still haunts the sector: the EU produces around 158 million hl of wine per year with an annual average consumption of 124 million hl[54]. The persisting imbalance between EU demand versus supply urges the expansion into international markets[55].

#### Vine planting authorization

Despite this imbalance, new rules for vine planting require Member States to authorise a 1% increase in the total area planted annually, which could result in a 16% increase in wine production area by 2030[56]. Even more perplexingly, the bulk of support focuses on increasing productivity rather than, as recommended by the European Court of Auditors, finding “*an appropriate policy mix*” to reduce the imbalance between supply and demand[57] Given the effects of excessive alcohol consumption on human health and resulting economic costs, many of the wine sector support provisions are inconsistent with public health objectives.

The CMO Regulation enables the attribution of EU funds to Member States “*to finance specific support measure to assist the wine sector*”[58]. Subsequent provisions spell out the measures eligible for support, including product promotion activities, vineyard conversion, grape destruction, insurances, investment and innovation assistance and by-product distillation support[59]. The EU budget for these support measures in the new CMO Regulation is set to €1.1 billion per year[60]. The chart below shows the expected national allocations per support measure for the years 2014–2018, amounting to a total of €6.2 billion of funding[61].

**Figure:**
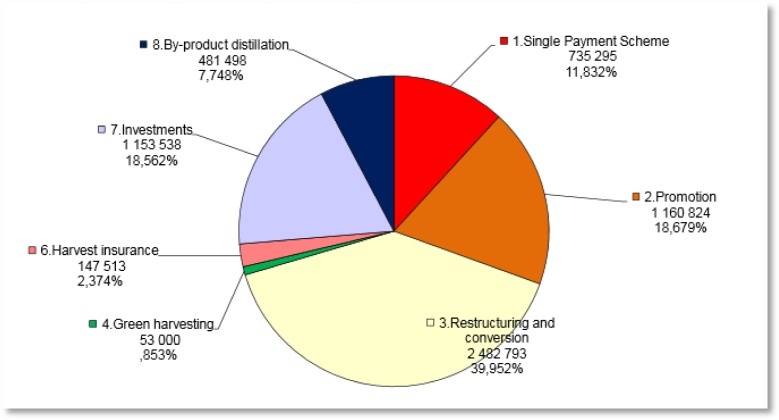
Wine National Envelopes 2014–2018 as reported in 2013 (European Commission)

#### Wine promotion

Any measure linked to wine promotion constitutes a (covert) way of promoting alcohol consumption and is incompatible with the notion of public funds for public goods. This also applies when promotion activities are formulated, as they currently are, in terms of campaigns to “*inform consumers about the responsible consumption of wine*” and foster the designation of origins and geographical indications schemes[63]. The European Court of Auditors found that promotional measures often constitute hidden operational subsidies as they replace the industry's own promotion activities[63]. If the EU is keen to enhance the visibility of its regional name protection schemes this should be carried out independently from wine promotion.

**Recommendation: End support for wine promotion measures.**

#### Investment support

Likewise, there is no justification to support the wine sector in making “investments in processing facilities and winery infrastructure, as well as marketing structures and tools” or “investments aimed at the development of new products, processes and technologies”[64]. The European Court of Auditors is also of the opinion that separate investment provisions for the wine sector are out of place as these measures are covered by the Rural Development Regulation[65].

Singling out wine producers for such support is unjust. These measures absorb valuable public money which could be used instead for promoting the production of foods essential for health, such as vegetables, fruits, pulses, nuts, and not alcohol whose harmful consumption represents a drain on the European economy.

#### Insurance support

Similarly, there is no justification for wine growers benefitting from insurance support through mutual funds and harvest insurances[66]. These risk-reduction measures are inappropriate considering that the negative health impacts of wine production are fully externalized.

#### Grape destruction

Support for the destruction or removal of grape vines in order to reduce yields to zero (‘green harvesting’) is not a structural measure but simply a way of managing oversupply[67] Support for by-product distillation[68], fortunately less damaging than the now abandoned support for the forced distillation of low quality wines[69], aims to reduce operational costs of wine production and should have no place in a health-consistent CAP. Particularly given that the measure's prime reason for existence was to have a positive effect on wine quality, which is according to a European Commission assessment no longer obvious[70].

**Recommendation: End preferential treatment for the wine sector. Phase out support for investments, innovations, harvest insurances, mutual funds, “green harvesting” and by-product distillation of wine production.**

#### Vineyard conversion

Support for vineyard conversion and restructuring largely aims at replacing older vineyards with newer, more productive ones in order to “*increase the competitiveness of wine producers*”[71]. The vineyard conversion measure should support conversion away from wine production or into the production of higher quality wines. This would allow some producers to exit the sector while improving the competitive position of those who remain. Such a measure would include assistance for small and medium-sized farm enterprises to diversify into new business models, like assistance in building local supply chains, enhancing wine quality, venturing into agro-tourism or cultivating other products. For instance, replacing vineyards with agroforestry systems could improve landscape quality and not impair environmental benefits, like carbon sequestration and erosion prevention, while offering a remunerative way of diversifying and ‘greening’ economic activity.

**Recommendation: Transform the provision for vineyard conversion away from quantitative productivity support towards qualitative assistance for producers to diversify into new business models.**

#### The right policy mix

The EU is the largest wine producer, exporter and consumer in the world. This is a source of pride, while a gradual decline in European wine consumption is beneficial to health and public budgets but, paradoxically, considered threatening[72]. This institutional mindset appears to legitimise public investment in the competitiveness of wine producers so as to fend off competition from third countries and ‘conquer’ international markets. Europe should mature away from this vision. Contributing to increase alcohol availability on the world market in full knowledge of the impact is a dubious objective. Instituting minimum unit pricing policies could be suitable measures to ensure European wines are not undercut by low cost competitors, while maintaining a non-discriminatory and health-beneficial policy regime.

The EU wine sector is heavily regulated and subsidised and while many commentators criticise this framework for being a waste of resources and creating vested interests, a right mix of regulatory incentives can achieve desired aims for the sector and society. Between 1953 and 1957 a package of measures in France, covering planting restrictions, subsidized uprooting of vines and obligations to replant with higher quality varieties, lead to a production decline of about 10% and improved wine quality[73].The grubbing-up measure introduced during the 2008 wine market reform delivered a reduction in supply of approximately 10 million hl. However, the support measure for restructuring of vineyards counterbalanced this decrease by enhancing yields[74].

Future policies should display a consistent mix of tools to ensure a sustainable supply-demand situation, pre-empting a health-compatible demand below the current level. Measures linked to (re)planting rights and vineyard conversions should again, as implemented successfully in the past, emphasise the transition from table wines towards higher quality produce[75]. More emphasis should also be put on maintaining the capacities of small- and medium scale wine growers who are far better equipped to take part in a leisure-based wine consumption culture, which is more in line with public health policy. A shift in business model from cheap table wines towards higher quality, higher value wines accompanied with lower levels of consumption can maintain or even enhance the total value of the wine market, while contributing to a substantial decline in liver mortality from alcohol[76].

**Figure:**
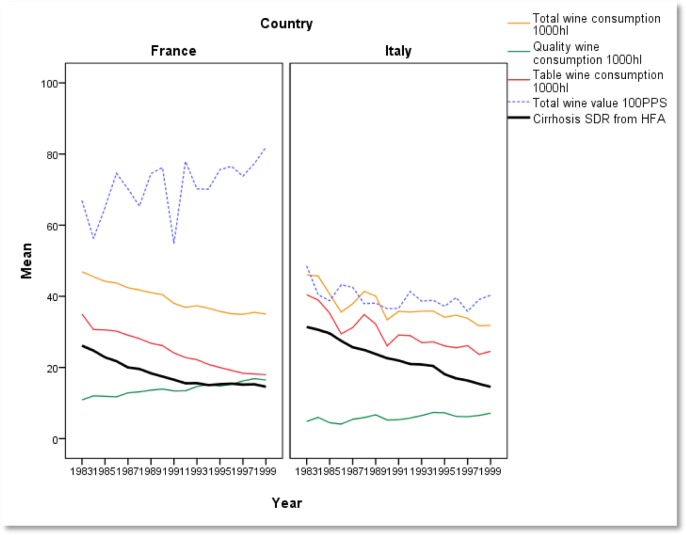
Wine consumption, liver mortality and wine market value (Nick Sheron, Univ. of Southamption)

**Recommendation: Amend the CMO Regulation to foster a transition towards new business models in the wine sector, with a focus on higher quality wine production, artisanal cultivation and income diversification.**

### No need to support hops

Hops is primarily used in beer production. The average EU citizen drinks 71 litres of beer per year, with country peaks at 144 litres and lows at 30 litres, down from an average 78 litres per person in 2008, but still among the highest levels in the world. Beer exports are on the rise[77]. Europe produces 60% of the world's hops, with Germany responsible for 60% of European production, or 1/3 of the global hops cultivated area. Germany is also one of the hubs in the international hops trade[78].

EU support for the hops sector was largely decoupled during the previous CAP reform[79], but now the Direct Payments Regulation makes hops eligible for the provision of voluntary coupled support[80]. Also, article 58 of the CMO Regulation provides for EU aid to producer organisations in the hops sector, singling out Germany as recipient of €2,277,000 per year. Given the central role of Europe and particularly Germany in the hops sector, there is no justification to continue this support. Reopening the possibility to apply coupled support to a negative health-sensitive product is a step in the wrong direction.

**Recommendation: remove any specific support for the hops sector from the CMO Regulation and remove hops from the voluntary coupled support option in the Direct Payments Regulation.**

### End all subsidies for tobacco cultivation

Tobacco has no other widespread commercial use than to be processed into tobacco products, killing 700,000 people in Europe each year[81]. The EU has managed to make significant progress on tobacco control and sizeable funds are now spent on smoking prevention and cessation throughout Europe. Any form of support to tobacco cultivation can therefore only be seen as a clear inconsistency.

Coupled support for tobacco production was nevertheless widely applied in the EU. In 2000 2.3% of the CAP and 1.09% of the total EU budget was spent on tobacco subsidies[82]. Despite vast investments of resources, the policy failed in its objective to promote a transition away from the cultivation of low-quality tobacco[83]. In the 1990s around two-thirds of EU produced tobacco was of low commercial value sold at minimum prices outside the European Community, while around 70% of EU's manufacturing needs were met by imports. With only about 55% of the subsidy granted being available as support for farm incomes, the remainder covering inputs purchases like seeds, fertilizers and other production factors, it would have been more cost-effective to simply grant income support to farmers without requiring them to produce an undesired product[84]–[86]. A profitability study suggested that without coupled support tobacco production would not be competitive compared to other crops, further questioning the subsidy policy[87].

At present, tobacco production is decoupled and no voluntary coupling is allowed[88]. However, in 2013 tobacco growers in five EU countries still received more than €83 million as additional support to improve crop quality. This scheme has only expired in 2014[89].

Tobacco farming remains part of the Direct Payments regime, which does not exclude the option that historical levels of support still partly determine current payments[90]. Tobacco farmers can furthermore benefit from support under the Rural Development chapters. Given the special case of tobacco, the most consistent, transparent and responsible way forward is to remove all European public funding for this crop. Instead the freed resources should be earmarked to assist tobacco farmers to diversify into other types of production, such as vegetables. This could be done in the framework of the Rural Development Regulation. This is a fair deal for farmers, public health and public budgets alike[91].

**Recommendation: Definitively exclude tobacco cultivation from any form of direct support through the Direct Payments Regulation and exclude tobacco farming from participation in any investment and other measures in the Rural Development Regulation. Released funds should be earmarked to support farmers diversify into other types of production.**

### No cheap sugar

Strategic aim: align the CAP with efforts to promote sustainable diets

The average European eats too much sugar. European adults take between 7–17% of their dietary energy from sugar and children even more – up to 25% in some countries[92]. The WHO recommends reducing free sugars intake to a maximum of 10% of total energy intake and preferably to no more than 5% (roughly 25 grams or 6 teaspoons per day)[93]. Excessive sugar consumption contributes to dental caries, overweight and obesity and other non-communicable diseases[94], potentially hampers memory and learning[95] and may induce depression in adolescents[96],[97]. Sugar has no nutritional value apart from the energy it provides.

Similar to wine, the EU sugar sector has known a long history of protectionism and faced significant levels of overproduction[98]. High sugar prices facilitated the growth, profitability and power of the European sugar industry[99]. In 2006 a substantial restructuring of the sugar sector saw an almost 25% decline in the volume of production quotas, the lowering of guaranteed prices and elimination of export subsidies[100]. The current system of quotas and market interventions, covering both sugar from sugar beet and isoglucose (also known as glucose-fructose syrup and made from either wheat or maize) is set to expire in 2017, largely liberalising the sugar market[101].

The European Commission predicts a substantial decrease in the price of sugar after abolition of the current quota regime[102]. A detailed study carried out by the Joint Research Centre (JRC) into a post-quota future models a drop in beet sugar prices between 15–24%, with an 18–19% decrease considered most probable[103],[104]. Human sugar consumption—euphemistically called ‘consumer surplus’ – is estimated to slightly increase. While this may not sound alarming, in reality it signals an unhealthy trend. In health terms sugar consumption must go down and any increase, however slight, will translate into a higher disease burden and increased healthcare spending. Lower sugar prices may furthermore discourage current efforts to reduce sugar content of processed foods though so-called “product reformulation”. A reduction in price may also have a more than marginal effect on sugar consumption. A French study suggests that a 36% sugar price decrease could enhance free sugar intake by 124g per person per year through sugary beverages only[105].

The JRC report presents several scenarios reflecting different assumptions about the expansion of isoglucose on the market for sweeteners. If isoglucose expands, sugar consumption may actually decrease. Unfortunately, isoglucose has the same health disadvantages as sugar and therefore, whichever way the isoglucose market reacts to quota abolition; the results will not be beneficial to public health[106].

Moreover, the abolition of quotas will probably be incoherent with EU development policy objectives. The EU is a net importer of sugar. The main source of imports at the moment are the least developed countries (LDCs) for whom raw sugar cane export constitutes a sizeable part of the economy and an important source of foreign exchange earnings[107],[108]. After quota abolition EU production is expected to substitute for much of these imports, while remaining import needs will be met by low-cost countries like Brazil[109].

In the current CAP sugar production is, quite illogically given the EU Member Stateś health recommendations, eligible for national voluntary coupled support[110]. During 2015 almost €200 million of support was coupled to maintaining sugar production levels[111].

**Recommendation: Maintain a sugar quotation system to prevent sugar prices from dropping. The sweetener market should not be fully liberalised as long as no EU-wide regulatory framework exists to internalise health costs into the price of artificially sweetened products, e.g. through fiscal measures.**

**Recommendation: Exclude sugar from the voluntary coupled support option in the Direct Payments Regulation.**

### Rebalance the supply of livestock products

Meat consumption in Europe is twice and dairy consumption three times the global average. For the maintenance of a healthy diet, the WHO recommends to reduce saturated fat intake to less than 10% of total energy intake[112]. The average European clearly exceeds this recommendation, primarily due to the excessive consumption of animal products[113].

**Figure:**
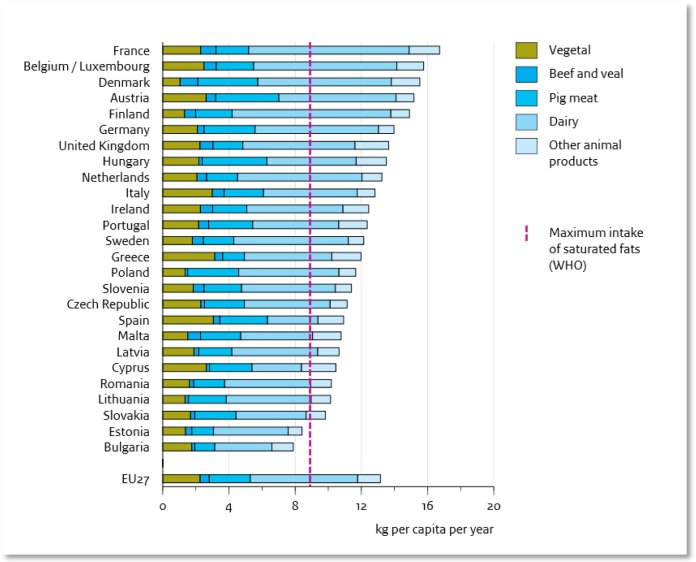
Intake of saturated fats in the EU27, 2007 (Netherlands Environmental Assessment Agency)

It is widely recognised that high levels of intake of animal products can lead to obesity, diabetes and cardiovascular diseases, which is also admitted by the European Commission[114]. The International Agency for Research on Cancer, an agency of the WHO, classified processed meat as carcinogenic to humans and red meat as probably carcinogenic[115]. The World Cancer Research Fund (WCRF) recommends limiting intake of processed meats including ham, salami, bacon and sausages[116],[117]. Animal farming is furthermore responsible for numerous negative ecological impacts.

Significant health and environmental benefits can be expected from lower red meat and dairy intake. Investigators examined the effects of a 50% reduction in the intake of beef, dairy, pork, poultry and eggs, under the assumption that reduced consumption would proportionally decrease production[118]. The results of this modelling exercise showed:

40% reduction in saturated fat intake, bringing saturated fats closer to levels recommended by the WHO;

Red meat consumption close to the maximum level recommended by the WCRF;

decrease in EU greenhouse gas emissions by 19–42% (dependent on the scenario and without capturing the substantial greenhouse gas reduction effects of decreased soya imports from South America which would drop by 75%);

23% fall in per capita use of cropland for food production, allowing among others for more extensive livestock farming;

40% drop in reactive nitrogen emissions. (Excess nitrogen emissions cost the EU €70–320 billion annually, of which 60% are related to health costs, averaging at €150–750 per person per year[119].)

if energy intake is reduced as a whole and partly substituted by vegetables and fruits, rather than only cereals as projected in the study, the health benefits could be even higher.

**A historical perspective on meat and milk**

Historically, the CAP employed various support measures geared at sustaining high prices for meat and milk products or making payments based on the number of animals kept on-farm[120]. Measures included floor prices, subsidized buying-up of surpluses (intervention buying), private storage aid, premiums per head and export subsidies. Even though such measures have ostensibly led to transfers from consumers to producers, suggesting that without subsidies consumers could have received animal products at a lower price, meat consumption in Europe more than doubled since 1961[121]. This jump in consumption of animal products was however insufficient to absorb the even more dramatic increases in supply.

Structural overproduction has resulted in much creative thinking about schemes to utilize surpluses at the least possible financial cost. A European Commission paper from 1980 addressing the “*intractable surplus problem*” in the milk sector gives a revealing insight into this thinking[122].

“In the case of skimmed-milk powder, extra sales have been achieved at the expense of vegetable protein, requiring a subsidy that may go up to 85% of the price paid for the powder by the intervention agency. …. For butter the problem is more difficult. … Nevertheless, the European Community has in recent years followed a policy of giving priority to its own consumers, and the quantities of butter to which subsidies have been paid to the internal market have been significantly greater than the quantities exported.”

Put bluntly – the CAP has been force-feeding European citizens with its fatty surpluses.

#### Voluntary coupled support

Recent CAP reforms have notably improved the situation compared with these historical excesses. However, greater opportunities remain to improve the CAP in terms of its livestock measures. The current CAP, in a regressive step, has allowed Member States to grant voluntary coupled support to milk and milk products, sheep meat, goat meat and beef and veal (but not pig meat)[123]. In fact up to 8% of total direct payments may be granted as coupled support. By way of derogation, this share can go up to 13% plus an additional 2% if used for protein crop production[124]. As a result, up to €6.3 billion annually could be coupled to production[125], while €4 billion is expected in the 2016 budget[126].

The condition for granting coupled support is that the sector in question must be “*particularly important for economic, social or environmental reasons*” and that it undergoes “*certain difficulties*”. Support may only create an incentive “*to maintain current levels of production*”, suggesting the measure is there for targeted reasons only. However, although Member States need to notify the Commission of their decisions to apply coupled support, nobody will actually evaluate this. In fact, pretty much any sector can be regarded as ‘particularly important’ for a specific region and considered to be undergoing ‘certain’ difficulties, especially in a volatile market. Also it is quite difficult to establish beforehand whether the application of coupled support will not encourage production beyond current levels[127].

Based on the notifications made to the European Commission in 2014 (see figure below), around 75% of coupled support is directly earmarked for livestock (42% beef and veal, 20% milk and dairy, 12% sheep and goatmeat) and an additional 10% for protein crops used in animal feed, totalling at around €3.3 billion in 2015[128].

**Figure:**
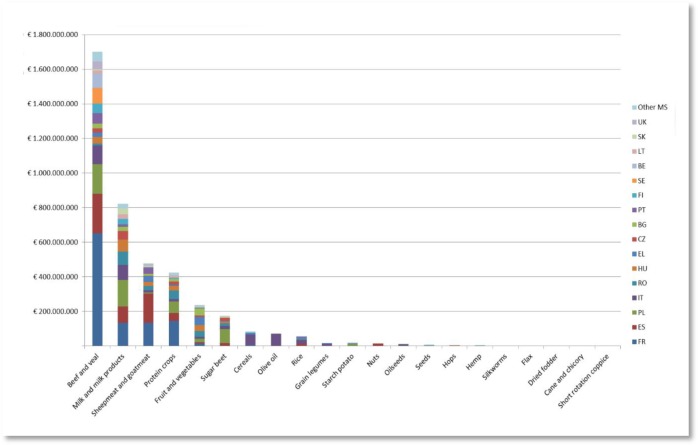
Voluntary coupled support: sectors supported (European Commission)

EPHA is sensitive to the aim of supporting rural communities where (extensive) livestock keeping is important to maintain their socio-economic and cultural fabric and to help perpetuate permanent grasslands. However it is doubtful whether the voluntary coupled support measure in its present format is sufficiently targeted to achieve this aim. The European Court of Auditors, in its audit of the suckler cow premium and sheep and goat aid schemes, appears to have similar concerns and found that these measures “*without explicit and sufficient targeting provisions … may have the effect of subsidizing other, more intensive animal farming methods*”. Furthermore, it found that this aid was not targeted to the most vulnerable needy regions, but was diluted by being distributed over the entire Member State[129]. Unfortunately there is little reason to believe the coupled support provision in its current format will deliver anything better.

Moreover, it is worth noting that according to the Regulation, “*any coupled support granted*” should be “*consistent with other Union measures and policies*”[130]. It does not appear that the proposed coupled support measures were adequately assessed on their likely health or environmental impacts.

**Recommendation: Remove all livestock products from the voluntary coupled support option in the Direct Payments Regulation. These funds should be re-allocated to targeted programmes under the Rural Development Regulation in order to support regions in which livestock keeping is vital for socio-economic reasons, maintains permanent grasslands and causes no environmental harm. Funds should likewise be used to assist farmers diversify into other forms of production.**

#### School milk scheme

The EU grants aid to the distribution of milk and milk products to children in school establishments through the so-called “School milk scheme”[131]. At the time of writing the school milk scheme is being amended and merged with the “School fruit and vegetables scheme”. A legislative package is being negotiated between the European Parliament, Commission and Council[132]. So far this new scheme is expected to allocate an additional €20 million per year for milk measures, bringing the total budget for support to milk products to €100 million annually. Member States have the flexibility to increase this budget by transferring up to 10% of funds from the fruit and vegetables scheme. At the same time, so far in the new measure, 10–20% of the total budget must be allocated to educational measures to enhance children's understanding of food and farming.

Whether the consumption of milk and milk products needs to be promoted is an ambiguous question. In the first place, the milk and meat sectors are complementary, with the former supplying ‘expired’ milk cows as well as calves to the latter. Secondly, European consumption of milk products is already higher than anywhere in the world. At the same time, the consumption of milk is slowly decreasing, which could justify a measure to keep children familiar with the product and possibly replace soft drinks. On the other hand, the consumption of cheese, also supported by the scheme, is on the rise[133].

**Figure:**
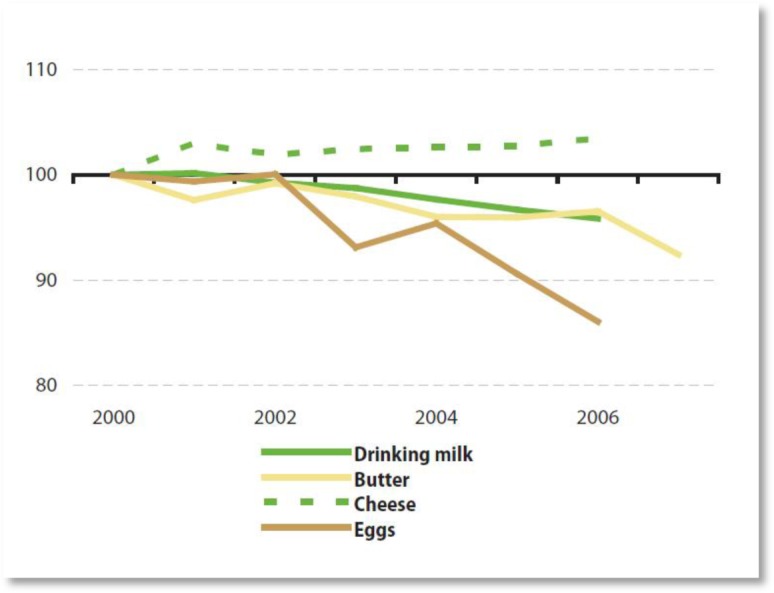
Consumption of milk products and eggs (Eurostat)

Milk contains different micronutrients, especially calcium, as well as protein and saturated fat (in whole milk). Milk is an accessible food for consumers that can form part of a healthy diet, but the quantity consumed varies dramatically from individual to individual[134]. Due to recommendations to reduce saturated fat intake, lower fat varieties of milk are recommended in Member Stateś national dietary guidelines with the possible exception in young children whose growth and development may benefit from full fat milk[135].

If the School milk scheme remains, an adequate nutrient profiling model should be applied to ensure that milk, yoghurts, cheese and other milk products do not exceed maximum salt, saturated fat and sugar levels. An obligation to choose products on the basis of health, environmental and ethical criteria, as well as giving priority to short supply chains, should be at the core for receiving EU co-financing. This scheme should not be regarded as either social assistance or as an additional outlet for “dumping” excess milk, but should primarily be an educational measure to reconnect children with food, food production and agriculture. Therefore, it is not the price-quantity, but the price-quality relationship that should be the core of the product distribution criteria.

**Recommendation: The new School milk scheme should apply nutrient profiling to determine which milk products, according to levels of salt, saturated fat and/or sugar, may be distributed in schools. Only products that conform to health, environmental and ethical criteria, as well as products from the region and/or using short supply chains, should receive EU co-financing.**

#### Market intervention

The CAP through the CMO Regulation maintains the possibility to intervene in the market by buying-up surplus beef, veal, butter and skimmed milk to protect market prices from collapsing. These products bought under public intervention may be “*disposed of*” by making them available to the most deprived[136]. Aid for private storage can be granted for beef, butter, cheese, skimmed milk, pig meat, sheep meat and goat meat[137]. In 2015 private storage was activated in the pig meat sector, temporarily taking 64.000 tons of pork off the market[138]. In the milk sector both private storage aid and intervention were activated in 2014. At the time of writing 108,652 tonnes of butter and 40,045 tonnes of skimmed milk powder were offered for private storage and 1,176 tonnes of milk powder for public intervention[139].

Export refunds are still allowed for beef and veal, milk and milk products, pig meat, eggs and poultry meat[140]. However these are no longer considered to be ‘regular’ market policy mechanisms, but to be activated only in case of “*market disturbances caused by significant price rises or falls*” or other circumstances significantly disturbing markets which are likely “*to continue or deteriorate*”[141]. Furthermore, exceptional market support measures can be taken for beef and veal, milk, pig, sheep, goat, egg and poultry in case of both animal disease outbreaks and loss of consumer confidence due to public, animal or plant health risks[142].

Market intervention measures can be justified for emergency situations where farmers' livelihoods are put at risk. More problematic is the context within which these intervention measures are embedded. The recent abolition of milk quotas and increased export-orientation of the livestock sector are enhancing farmers' susceptibility to volatility and risks[143]. Dependence on exports has shown its ugly face with the Russian ban on EU agricultural imports and the lower-than-expected demand from China[144]. Pork and beef prices are low while milk prices are falling threatening many small to medium sized farmers with bankruptcy[145]. High production levels, without sufficient demand, international competition, and increasingly powerful retailers in the EU have set the scene for recent farmers' protests, like those in France, Belgium, Brussels and the UK. Farmers' representatives, many of whom were pushing for the abolition of milk quotas, now lament the critical situation and are demanding public support through export restitutions, storage aid, increased intervention prices and the non-collection of milk super-levies[146].

When the interrelationships between supply, demand and trade are so maladjusted, market intervention measures can only “pay lip service” to a failing system and will perpetuate rather than solve the CAP´s problems. The majority of farmers want fair prices, rather than subsidies[147]. But prices, to be truly fair, must also reflect the externalities to the environment, public health and animal welfare. The most logical solution is a transition towards a food and farming system where consumers pay higher prices for EU primary products; farmers keep less livestock for better prices; and where farmers receive support through the Rural Development pillar to reduce input costs through innovation, to improve environmental performance, to increase animal welfare, to maintain permanent grasslands, to promote short supply chains and so forth.

A scenario where both livestock rearing and animal product consumption is decreased will better deliver these welfare and environmental benefits while also being more economically beneficial to farmers. In addition, human health benefits arise from extensively reared animal products which tend to have better nutritional profiles due to lower saturated fat content, increased levels of omega-3 fatty acids and an improved balance of vitamins and minerals[148]–[150]. Similarly, milk from grass-fed cows has several health benefits compared to milk from cows being fed feedlot diets, including a better fatty acids profile[151],[152]. Furthermore, improved welfare and lower stocking densities enhance immunity and reduce probability of disease outbreaks, reducing the need for antibiotics and so lowering the threat of antibiotic resistance as well as the transmission of other zoonotic diseases[153].

**Recommendation: Develop a transition plan, including through economic incentives, towards a lower livestock product consumption and production future which ensures benefits to public health, the environment, farmers' incomes and rural communities.**

### Boost vegetables and fruit production & consumption

The WHO recommends a minimum intake of 400g of vegetables and fruit (V&F) per day[154]. Regardless of methodological difficulties in measuring consumption, there are firm indications that the average European does not eat enough[155]. The latest consumption monitor by Freshfel puts average V&F consumption in Europe at 342 g/capita/day, a 1.9% decrease compared to the 2008–2012 average. 22 of the 28 EU Member States are below WHO recommendations[156]. V&F consumption by materially deprived households is far below average, with about 10% of EU households unable to provide children with fruit on a daily basis[157]. More than 2.5–3.9% of the burden of disease in Europe in 2004 is attributable to low V&F consumption[158].

#### School fruit scheme

The EU “School fruit and vegetables scheme” (School fruit scheme) promotes vegetables and fruit consumption with schoolchildren[159]. This scheme is being amended and merged with the School milk scheme through a package currently negotiated between the European Parliament, Commission and Council. The proposed new “Aid scheme for the supply of fruit and vegetables, bananas and milk in the educational establishments” (School milk and fruit scheme) mentions the contexts of declining V&F consumption and rising childhood obesity as well as the aim to promote “*healthy eating habits and the consumption of local products*”. The annual budget for the vegetables and fruit section of the School milk and fruit scheme has increased from €90 million to €150 million and can be further enhanced by transferring up to 10% of the budget from the school milk section (see above). 10–20% of the total yearly budget of the combined aid scheme, which is €250 million, should be allocated for educational measures meant to “*reconnect children with agriculture and the variety of Union agricultural products*”[160].

The School fruit scheme has great potential and EPHA has consistently spoken out in its favour[161]. The Scheme also received overall positive evaluations, including by the European Court of Auditors[162],[163]. The main long-term value of the scheme is when children effectively discover and become accustomed to the variety of tastes and organoleptic qualities of unprocessed vegetables and fruits, building acceptance, especially of vegetables, at a young age and providing a knock-on effect for food choices throughout life. The scheme can achieve its full potential when both sourcing of vegetables and fruit and the educational elements, foreseen by this new measure, are integrated into one consistent activity.

When full use is made of local supply chains, visits to farms can be made to those particular places where the distributed products are cultivated, adding significantly to the connection with agriculture and realization of where food comes from. Allowing local specialities to be distributed as part of the educational element may provide a positive addition to childreńs realization of the diversity of agricultural outputs, but the inclusion of processed products must be limited[164].

**Recommendation: Refer to this scheme, which combines supply and demand measures and which has the potential to benefit both health, farmers and public budgets alike, as an example for other food policy initiatives.**

**Recommendation: Gradually increase the budget for this scheme, in line with increased take-up, and enhance co-financing rates for schools in economically deprived areas where vegetable and fruit intake is especially low.**

**Recommendation: The new School fruit scheme should contain more guarantees that EU funding is linked to a maximum effort from schools to ensure that the scheme will positively affect children's food choices later in life. This could be done, for instance, by integrating product sourcing and education into one coherent activity.**

#### Producer organisations

After reforms in 2007, EU support for the vegetables and fruit (V&F) sector is primarily channelled through producer organisations (PO's), membership of which is meant to improve the bargaining power of V&F producers in globalizing and increasingly concentrated supply chains[165]. POs implement operational programmes to plan production, improve product quality, boost commercial value, enhance promotion of V&F, improve environmental quality and manage crises[166]. In 2014 more than € 930 million was granted in support of POs, but the budget appears to be decreasing[167]. The policy was effective in enhancing the popularity of POs however substantial differences in organisational rates across Europe remain[168].

**Figure:**
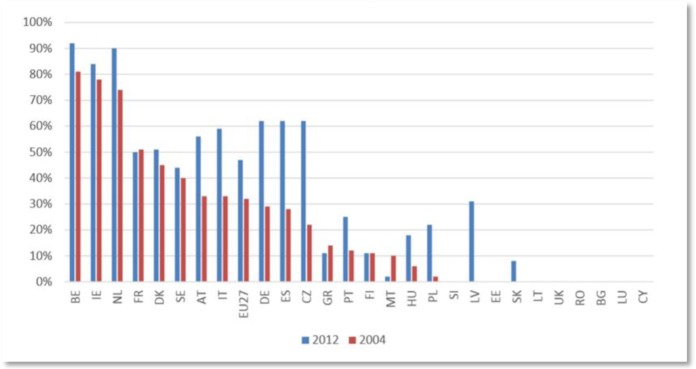
PO organisation rates compared across Europe (Wageningen University)

One of the public health criticisms of the old policies was their “withdrawal” (destruction) of V&F from the market. While such measures help to maintain producer prices, they simultaneously increase prices for consumers[169],[170]. Product withdrawal activities are still financed in the new CAP, but indirectly through ‘crisis prevention and management’ measures implemented by POs. These include market withdrawal and green- and non-harvesting measures. Withdrawn products may be, but not mandatorily so, earmarked for free distribution to charities and public institutions like prisons, schools, hospitals and old people's homes. Free distribution is 100% financed by the EU[171].

Any policy that has the outcome of increasing V&F prices for consumers is working against health. Prices for fresh V&F have been increasing while prices of highly processed foods have gone down. Technological improvements in the processing sector have decreased production costs per unit of output. Highly processed foods tend not to rely on quality farm products, but achieve added value through manufacturing processes combining relatively cheap ingredients enhanced by sugars, salts, fats and flavours[172].

The area for V&F production in Europe decreased by 6% in the period 2003–2010 and total production is slowly decreasing, even though total value remains the same[173],[174]. Although exports as well as imports are on the rise, Europe remains a net V&F importer[175],[176]. The decline in cultivated area and production, contrasted by the clear public health recommendation to increase V&F intake, is alarming and a clear example of where the current CAP fails to be consistent with health. The cost of insufficient fruit and vegetable intake can be estimated at €22.3 billion per year in its contribution to cardiovascular disease only[177]. If EU citizens were to consume 600g of vegetables and fruits per day, as recommended by Denmark, the risk of coronary heart disease would fall by almost 20%[178].

V&F production represents only 3% of total European cultivated area, but 18% of value added in agriculture[179]. Whereas if V&F production was increased this could enhance economic returns while also increasing employment opportunities, given the labour intensive nature of horticulture. Although POs are potentially able to enhance the commercial viability of horticultural producers, their main focus is marketing and not increasing availability of V&F to European citizens. Moreover other measures are needed to support those horticultural producers (60% of total) who are not members of POs.

#### Fostering production & consumption

However, increased horticultural production is only half of the equation and, without enhanced vegetable and fruit consumption, it is doomed to result in falling farmgate prices and more food waste. Vegetables and fruits should become better available to those groups in the population that need them most. A pilot programme in the US found that providing financial incentives to buy V&F for low-income households helped improve diets, rather than leading to more money being spent on junk food[180]. Likewise, young mothers participating in the UK “Healthy Start” program ate significantly more portions of vegetables and fruit and were more likely to meet micronutrient intake levels without vitamin supplements[181]. The Preparatory Action on fruit and vegetables consumption, aimed at increasing consumption of fresh vegetables and fruit in vulnerable population groups, supported by the European Commission is also a positive action in this regard[182].

At the same time, care must be taken not to overemphasize the quantity of production over quality. There is evidence that the amount of minerals in commonly grown crops has diminished over the last few decades[183]. Nutritional content of vegetables and fruit heavily depend on the used varieties. In apples, some of the most heavily cropped varieties, like Golden Delicious, have some of the lowest levels of vitamin C[184]. Also, increased V&F production must go hand-in-hand with the enhanced uptake of integrated pest management techniques[185].

**Recommendation: To analyse evidence and experiment with policy action with the aim of enhancing both production and consumption of vegetables and fruit in Europe. Such a strategy should be based on the premise that vegetables and fruit, including leguminous crops, merit positive discrimination at the stage of cultivation and in marketing. The strategy should also take account of the nutritional value and mineral content of vegetables and fruit, the need to reduce chemicals use, ensure adequate market opportunities for small- and medium sized non-PO members, and healthy occupational conditions for farm workers.**

### Promote products for diverse and healthy diets

The EU supports promotional campaigns for European agricultural products both within and outside Europe. The new 3-year programme published in 2015 has a budget of €130 million, half-financed by the EU. Around €20 million EU funding is for the promotion of meat and dairy products, €25 million for fresh fruits and vegetables, €13 million for olive oil and olives[186]. Other categories include wine, processed fruits and vegetables and organic products. In December 2015 new rules come into force which enhance the existing budget to €200 million per year, provide higher rates of EU co-financing and more focus on export promotion. All agricultural products are eligible except for tobacco[187],[188].

Product promotion should not centre on objectives such as enhancing competitiveness of the EU agricultural sector or raising “*awareness of the merits of the Union's agricultural products*”[189]. Instead this promotion should provide an opportunity to drive a transition towards more biodiverse, sustainable and healthy diets, rather than ‘conquering’ markets for the agricultural sector. Therefore, the primary focus of promotional efforts should be in the internal market rather than outside the EU which is associated more with re-distribution of excess production. Alcohol and livestock products should be excluded from receiving EU support for promotional activities as their enhanced consumption cannot be associated with healthier diets.

**Recommendation: Amend the Regulation on product promotion measures to focus exclusively on products whose enhanced consumption is likely to contribute to more biodiverse, sustainable and healthy diets. Products should include vegetables and fruit, pulses, nuts, whole grains, fish from aquaculture conforming to strict environmental, welfare and food safety standards, nutritious varieties of cereals etc. Product promotion should focus mainly on the internal market.**

### Transition towards forward-looking direct payments

Since the mid-1990s, direct payments have become core instruments in the provision of farm support. Today, direct payments represent around 70–75% of total CAP funding, or nearly €312.7 billion for the period 2014–2020 (amount includes expenditures for market measures). Direct payments were initially introduced as coupled payments linked to the levels of production – the higher the production, the higher the support – but were gradually ‘decoupled’[190],[191].

#### Convergence

In the new CAP, the main principle is that farmers receive payments per hectare of land, envisioning the ideal situation in which all farmers would receive an equal amount per hectare, regardless of the crop cultivated or number of animals held. This process of ‘convergence’ would in principle have to be completed by 2019, but many exceptions remain[192].

**Figure:**
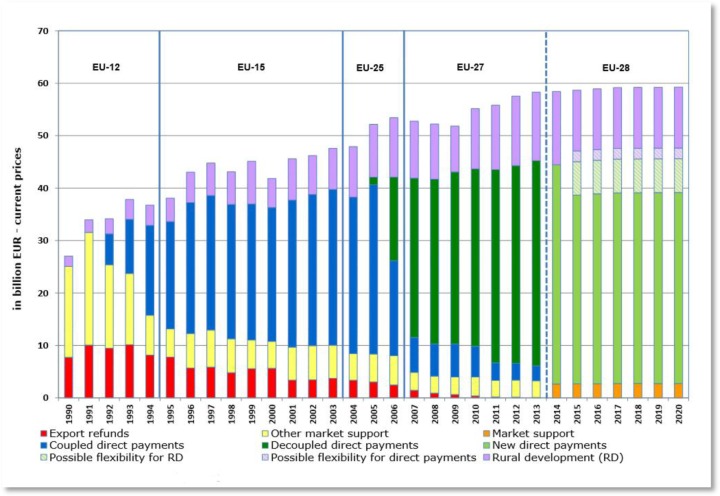
Evolution of CAP expenditures 1990 – 2020 (European Commission)

Member States may, as some did, opt for slower convergence rates and in doing so protect the interests of the often more intensive and large-scale farmers who have received high levels of support in the past[193]. Those levels of support are usually based on a historical reference, from the time that payments were not yet decoupled[194]. The possibility for new member states to apply the Single Area Payment Scheme until the end of 2020 may furthermore allow for hidden production linkages[195]. Likewise, Member States applying the Single Payment Scheme on regional level can continue doing so[196], even though doubts on the truly decoupled nature of this scheme have been raised[197]. So, unless full convergence takes place, the coupled payments from the historical CAP will still be influencing the relative profitability of different types of enterprises and so shape production decisions.

**Recommendation: Move to full convergence of payments as soon as possible, at the latest by 2020, to remove incoherent subsidy legacies from earlier versions of the CAP.**

#### Redistribution of payments

The CAP has been much criticized for its unequal distribution of subsidies. The often-quoted figure is that 80% of subsidies go to 20% of farms[198]. For individual countries more extreme inequalities are calculated, like Bulgaria and Romania where the top 1% of beneficiaries receive around 50% of direct support[199]. The new CAP reform introduced several options to reduce this misbalance. In addition to convergence, one option is the ‘redistributive payment’ which allows Member States to use up to 30% of their direct payment ceilings to give additional support to farms under the size of 30 hectares[200]. Another option is the ‘small farmers scheme’ which allows granting additional favours and exemptions to small farmers[201]. Furthermore, Member States are obliged to apply ‘degressivity’ by reducing the amount of direct payments exceeding €150.000 in a given calendar year by at least 5%. Optionally, this reduction can go up to 100%, establishing a true ‘cap’ on the total amount of subsidy each farmer or land owner can receive[202].

Although these additional options permit a system of positive discrimination for certain types of holdings, thus technically undermining the principle of convergence, they should be considered as positive tools to counterbalance the decline in farm numbers. Europe's ageing farming population has fallen by half over the last 25 years with a corresponding increase in farm sizes and decrease in employment opportunities in agriculture[203]. The exit of small farmers from agriculture may furthermore lead to rural unemployment and depopulation within rural areas in Member States with less-developed infrastructure[204],[205], as well as increased urbanisation with its corresponding problems[206]. Rural decline can manifest itself in ill-health, including mental problems, amongst the remaining populations[207]. The redistributive policy options may not be sufficient to counterbalance negative trends, also because they are mostly dependent on individual Member States' discretion.

Horticultural producers are also in need of another approach since they are negatively discriminated against by the CAP´s direct payment regime, based on payment per hectare, because their farm sizes tend to be far below the EU average[208]. Horticulture is responsible for only 3% of the total agricultural area in Europe. However, as discussed above, increased V&F production could contribute to: employment opportunities; increased added value; vibrant rural economies and slower rate of urbanisation; substituting imports and enhanced EU V&F availability which could help increase consumption.

**Recommendation: Introduce positive discrimination in the Direct Payments Regulation for small- and medium-size farmers, especially vegetables, fruit and pulses producers. Put an absolute cap on the total amount that each farmer or land-owner can receive.**

#### Environmental conditionality

‘Cross-compliance’, as well as the new ‘greening’ measures, are the so-called environmental standards which must be complied with in order to receive (part of) the direct payments[209]. Cross-compliance includes compliance with already mandatory pieces of EU legislation, like the Nitrates, Habitats and Birds Directives, animal welfare Directives, identification of animals Directives and Regulations, but also Good Standards of Agricultural and Environmental Conditions (GAEC). Greening measures, hotly debated even before the new CAP implementation started and now in the process of being ‘simplified’[210], centre on requirements for crop diversification, maintenance of permanent grasslands and ecological focus areas[211].

Agriculture produces significant externalities with impacts on: climate change; the quality of nature, biodiversity and landscapes; pesticide hazards; air quality; antibiotic resistance; other zoonotic diseases and so there is clear need to transition towards farming practices that drastically reduce agriculture's burden on both the planet's and human health. This transition includes periodically upgrading minimum standards for, e.g. animal welfare, nitrate emissions and pesticides, but also primarily by promoting innovative sustainable farming practices and remunerative business models, which are best achieved through rural development programmes. A forward-looking CAP would therefore shift from direct payments to rural development support, whose measures can be more targeted and capable of achieving specific aims.

**Recommendation: Maintain an effective, efficient and well-enforced system of environmental protection in which standards are upgraded in line with societal needs. Add the ‘Integrated Pest Management Directive’[212] to the list of cross-compliance measures.**

**Recommendation: Shift the financial focus of the CAP away from direct payments towards rural development. A forward thinking agricultural policy would have most (75%) of its funding distributed through rural programme-based payments (Pillar II) and much less (25%) through direct payments (Pillar I).**

### Support transformative rural development

The rural development pillar of the CAP complements direct payments and market measures “*to contribute to [the CAP's] objectives*”[213]. During negotiations for the 2014–2020 Multiannual Financial Framework, the rural development fund suffered from the largest budget decline—18% compared to only a 13% reduction for the direct payments and market measures. For the period 2014–2020 nearly €95.5 billion of EU support is set aside to co-finance rural development projects[214].

Member States can choose from a wide ‘menu’ of available measures to shape their rural development programmes[215]. Funding can be used to promote organic farming, which can be seen as a pioneering method for agro-ecological innovation. Agroforestry, which offers wide-ranging co-benefits including improved water and nutrient cycling, pest management, erosion prevention, enhanced production of nuts, climate change mitigation (according to one study, deployment of agroforestry to its full potential could sequester more than 1/3 of all EU greenhouse gas emissions)[216] and improved farm economies[217]. It allows farmers to be compensated for: reducing pesticide and fertilizer use; improving animal welfare standards; adopting mixed farming methods; and using more advanced cropping systems. Funding is also available for village renewal measures as well as additional support for farming activities in areas with natural constraints. Furthermore, actions like ‘cooperation’, ‘Leader’ and ‘European Innovation Partnerships’ can be used to establish bottom-up collaborative projects by farmers, researchers, SMEs and civil society groups.

Clearly, the rural development pillar has much to offer in terms of moving towards a CAP that delivers public goods for public money and allows farmers to innovate in a sustainable manner. However, at the same time, rural development funding through the ‘investments in physical assets’ heading, can also be used to finance the construction of housing for cattle, pigs and chickens as well as manure storage and processing facilities. Such investments, often used to facilitate the expansion of intensive livestock system, are funded by the public for up to 40% of investment costs[218]. Also, as shown by the European Court of Auditors, not all rural development measures are implemented and controlled effectively[219].

Nevertheless the rural development pillar offers the best available option to shape a CAP which promotes sustainable innovation in farming practices and business models, thriving rural areas, improved environmental performance and responsiveness to public health. Unfortunately, in addition to the greater reduction in the rural development budget, the new CAP has also allowed Member States to transfer funds away from rural development into their direct payments pillar. This is a clear reversal of the principles behind the previous CAP reforms which aimed to progressively increase the rate of so-called ‘modulation’ – i.e. the transfer of funds from the Direct Payments into the Rural Development pillar. Whereas the latest CAP allows up to 15% of national envelopes to be transferred either way from one pillar to the other, which some Member States actually did[220],[221].

**Recommendation: Transfer the CAP budget away from direct payments and market measures (Pillar I) towards rural development (Pillar II). A clear timeline must be set to have at least 75% of CAP funding channelled through rural development measures.**

**Recommendation: Conduct a thorough review of measures supported by the rural development fund in order to single out the key measures with greatest potential to contribute to sustainable socio-economic and technological innovation in agriculture and facilitate a transition towards a sustainable and health-compatible food system.**

**Recommendation: Increase co-funding rates for the key measures identified, while excluding funding from those measures that contribute to the proliferation of socially, environmentally and public health-harmful outcomes.**

**Recommendation: Include aquaculture as a farm diversification measure, accompanied by strict binding and enforced conditionalities regarding feed, medicine (antibiotic) use and pollution control. Fish offers human health benefits compared to consumption of livestock products and is more efficient in feed conversion.**

**Recommendation: Offer support for care farms, where farming activities are used as therapeutic practices for vulnerable groups of people especially with mental health benefits, as a measure eligible for rural development support.**

### There is no such thing as cheap food

This paper envisions a CAP that facilitates the transition towards diverse, sustainable and healthy diets. Sustainable eating patterns reflect public health, environmental, social and economic requirements[222]. This transition consists of a move towards more varied, healthy plant-based diets, the preference of quality produce over quantity, more agro-ecological agricultural methods, and fair prices for farmers. Europe needs a farming sector where farmers can feel secure to introduce new, sustainable agricultural practices and business models. This is difficult at a time when indebtedness and market volatility are high, while farm incomes are less than half the average wage in the majority of EU's regions[223].

Such a transition in EU agriculture will affect the relative price of certain foods and may have an effect on affordability. This even while most of the price addition actually occurs beyond the farm. Average EU household expenditures on food are less than 15% of total household expenditure, suggesting there is room for paying a higher price for some types of foods [224]. But averages conceal the sharp social inequalities that exist between countries in the EU and within countries, which are increasing[225]. One out of four EU citizens is at risk of poverty and for many poor households food represents a significant part of total expenditure, restricting their food choices[226],[227]. At the same time, it is the vulnerable socio-economic groups that are disproportionally affected by tobacco, alcohol and diet-based and environmentally induced ill-health[228],[229]. For instance, people from low income socio-economic groups are around twice as likely to become obese. Obesity is a major cause of premature mortality and healthy life years lost in this segment of the population[230].

While for an individual the price of food may matter, from a societal perspective cheap food does not exist. What is not paid at the counter is paid through tax, or not paid at all, leaving future generations to foot the rising bill for treatment of chronic diseases and ecological degradation. Dynamic societies and vibrant economies can only be sustained if they are underpinned with a healthy environment as well as a healthy population[231],[232].

A transition in agriculture should occur on the basis of the scientific evidence, rights, principles and basic societal requirements. Individual farmers and horticultural producers, as well as vulnerable socio-economic groups, need better incentives to bridge certain parts of this transition. This strengthens the argument for a coordinated approach to food and agricultural policy covering the entire supply and marketing chain, including international trade.
